# Endothelial-derived interleukin-6 induces cancer stem cell motility by generating a chemotactic gradient towards blood vessels

**DOI:** 10.18632/oncotarget.22225

**Published:** 2017-11-01

**Authors:** Hong Sun Kim, Yu-Chih Chen, Felipe Nör, Kristy A. Warner, April Andrews, Vivian P. Wagner, Zhaocheng Zhang, Zhixiong Zhang, Manoela D. Martins, Alexander T. Pearson, Euisik Yoon, Jacques E. Nör

**Affiliations:** ^1^ Department of Restorative Sciences, University of Michigan School of Dentistry, Ann Arbor, MI, USA; ^2^ Department of Electrical Engineering and Computer Science, University of Michigan, Ann Arbor, MI, USA; ^3^ Department of Oral Pathology, Universidade Federal do Rio Grande do Sul, Porto Alegre, RS, Brazil; ^4^ Department of Periodontics and Oral Medicine, University of Michigan School of Dentistry, Ann Arbor, MI, USA; ^5^ Department of Internal Medicine, University of Michigan Medical Center, Ann Arbor, MI, USA; ^6^ Comprehensive Cancer Center, University of Michigan, Ann Arbor, MI, USA; ^7^ Department of Biomedical Engineering, University of Michigan College of Engineering, Ann Arbor, MI, USA; ^8^ Department of Otolaryngology, University of Michigan School of Medicine, Ann Arbor, MI, USA

**Keywords:** cancer stem cells, epithelial-mesenchymal transition, migration, head and neck squamous cell carcinoma, metastasis

## Abstract

Recent evidence suggests that the metastatic spread of head and neck squamous cell carcinomas (HNSCC) requires the function of cancer stem cells endowed with multipotency, self-renewal, and high tumorigenic potential. We demonstrated that cancer stem cells reside in perivascular niches and are characterized by high aldehyde dehydrogenase (ALDH) activity and high CD44 expression (ALDH^high^CD44^high^) in HNSCC. Here, we hypothesize that endothelial cell-secreted interleukin-6 (IL-6) contributes to tumor progression by enhancing the migratory phenotype and survival of cancer stem cells. Analysis of tissue microarrays generated from the invasive fronts of 77 HNSCC patients followed-up for up to 11 years revealed that high expression of IL-6 receptor (IL-6R) (p=0.0217) or co-receptor gp130 (p=0.0422) correlates with low HNSCC patient survival. We observed that endothelial cell-secreted factors induce epithelial to mesenchymal transition (EMT) and enhance invasive capacity of HNSCC cancer stem cells. Conditioned medium from CRISPR/Cas9-mediated IL-6 knockout primary human endothelial cells is less chemotactic for cancer stem cells in a microfluidics-based system than medium from control endothelial cells (p<0.05). Blockade of the IL-6 pathway with a humanized anti-IL-6R antibody (tocilizumab) inhibited endothelial cell-induced motility *in vitro* and decreased the fraction of cancer stem cells *in vivo*. Notably, xenograft HNSCC tumors vascularized with IL-6-knockout endothelial cells exhibited slower tumor growth and smaller cancer stem cell fraction. These findings demonstrate that endothelial cell-secreted IL-6 enhances the motility and survival of highly tumorigenic cancer stem cells, suggesting that endothelial cells can create a chemotactic gradient that enables the movement of carcinoma cells towards blood vessels.

## INTRODUCTION

Head and neck cancer is the sixth most common cancer worldwide [1, 2]. HNSCC comprises 90% of all tumors of the head and neck region. The overall 5-year survival rate is 80% in patients with early stage disease, but the rate drops to 20-50% in late stage patients [3]. Approximately half of the late stage patients develop locoregional or distant metastasis, which significantly lowers the survival rates of head and neck cancer patients [4]. The understanding of mechanisms driving the invasive behavior of tumorigenic HNSCC cells is critical for the development of a mechanism-based therapy that prevents tumor dissemination.

Cancer stem cell theory postulates that the current failure in cancer treatment is due to our inability to target tumor cells that are resistant to radiotherapy and chemotherapeutic agents [5]. Cancer stem cells are uniquely tumorigenic and have the ability to self-renew and differentiate. Prince and collaborators first identified head and neck cancer stem cells using CD44 expression alone [6]. Subsequent report showed that ALDH activity-based cell isolation selects for cancer stem cells in HNSCC [7]. Our group used the two markers together and found that the ALDH^high^CD44^high^ cells are uniquely tumorigenic cancer stem cells in HNSCC [8].

Like normal stem cells, cancer stem cells reside in a niche microenvironment to survive and protect the self-renewal ability [9]. Our group has shown that head and neck cancer stem cells reside in perivascular niche [8]. In theory, proximity between cancer stem cells and blood vessels makes it easy for the cancer stem cells to migrate and invade into blood vessels to initiate metastasis. Endothelial cells secrete multiple cytokines that affect the behavior of tumor cells. For example, endothelial cell-secreted CXCL8 increases the frequency of local recurrence in preclinical models of HNSCC [10]. In addition, endothelial cell-secreted factors induce epithelial-mesenchymal transition (EMT) and migration in HNSCC [11], suggesting the endothelial cell-tumor cell interaction plays a critical role in cancer progression. However, the effect of endothelial cell-secreted factors on the invasive behavior of the highly tumorigenic cancer stem cells remains to be determined.

IL-6 is a pro-inflammatory cytokine that activates JAK/STAT3 pathway. IL-6 level has been correlated with tumor progression in multiple cancer types [12–15]. A prospective cohort study found that pretreatment serum IL-6 level was a predictive marker for recurrence rate and overall survival of HNSCC patients [16]. Independent studies showed that tumor cells acquire metastatic potential through IL-6/STAT3 pathway [17, 18]. IL-6 is secreted by many different cells, including T cells, B cells, monocytes, endothelial cells, fibroblasts, and some tumor cells [19]. Upon inflammatory stimulation, endothelial cells secrete high levels of IL-6 [20]. We previously have reported that tumor-associated endothelial cells lining tumor blood vessels express more IL-6 than the tumor cells themselves [21]. However, the role of endothelial cell-secreted IL-6 on migratory behavior of head and neck cancer stem cells have not been investigated.

Here, we evaluated the significance of endothelial cell-secreted IL-6 on head and neck cancer stem cell motility and the therapeutic potential of targeting IL-6 pathway in HNSCC. We observed that expression of IL-6R or its co-receptor, gp130 in the invasive front of primary HNSCC correlated with poor overall patient survival. Endothelial cell-secreted IL-6 induced EMT and enhanced migration in head and neck cancer stem cells. Collectively, these results demonstrate that endothelial cell-secreted IL-6 induces a migratory phenotype in head and neck cancer stem cells and suggest that the progression of HNSCC towards metastasis or recurrence might be prevented or delayed by therapeutic blockage of the IL-6 pathway.

## RESULTS

### Expression of IL-6R or gp130 correlates with HNSCC patient outcome

**Figure 1:**
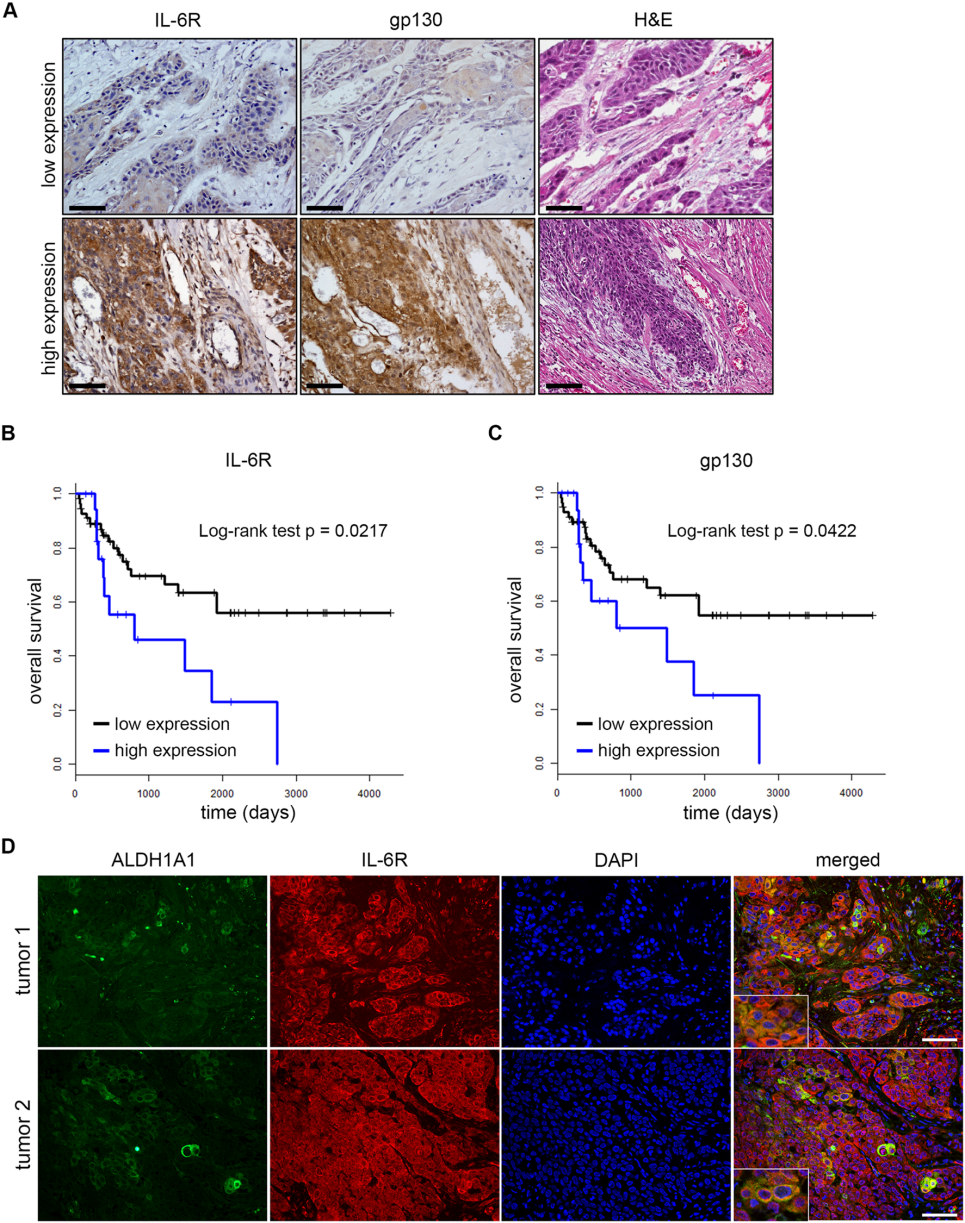
Expression of IL-6R or gp130 correlates with HNSCC patient outcome **(A)** Representative immunohistochemistry staining of low and high expression IL-6R and gp130 and corresponding hematoxilin and eosin (H&E) staining. Scale bars=100 μm. **(B)** and **(C)** Kaplan-Meier curves of oral squamous cell carcinoma patients over IL-6R and gp130 expression respectively. **(D)** Immunofluorescence staining of ALDH1A1, IL-6R, and DAPI in invasive fronts of two primary patient tumors used in TMA. Scale bars=100 μm.

To evaluate the correlation between tumor IL-6R and gp130 and the outcome of patients, we used a tissue microarray (TMA) containing 77 specimens prepared from the invasive front of HNSCC tumors (Supplementary Figure 1). Two oral pathologists blinded for experimental conditions scored independently these specimens as low (score ≤4) and high (score >4) IL-6R or gp130 expression based on IHC staining (Figure 1A). We found that high IL-6R expression in the invasive front of the tumor correlated with poor overall survival (log rank test, p=0.0217; Figure 1B). To control for other known prognostic variables, we performed multivariate regression analysis, and IL-6R maintained significant discriminating ability for overall survival (Cox PH model, p=0.0128) in a model including age, tobacco use, advanced stage at diagnosis, race, sex, and alcohol. gp130 expression also had strong correlation with the patient overall survival (log rank test, p=0.0422; Cox PH model, p=0.0243) (Figure 1C). Immunofluorescence staining showed that IL-6R was strongly expressed by most cells at the invasive fronts of HNSCC (Figure 1D). As expected, the ALDH1A1-positive cancer cells are more rarely observed, which is consistent with the cancer stem cell hypothesis.

### Therapeutic inhibition of the IL-6 pathway decreases the cancer stem cells fraction

**Figure 2:**
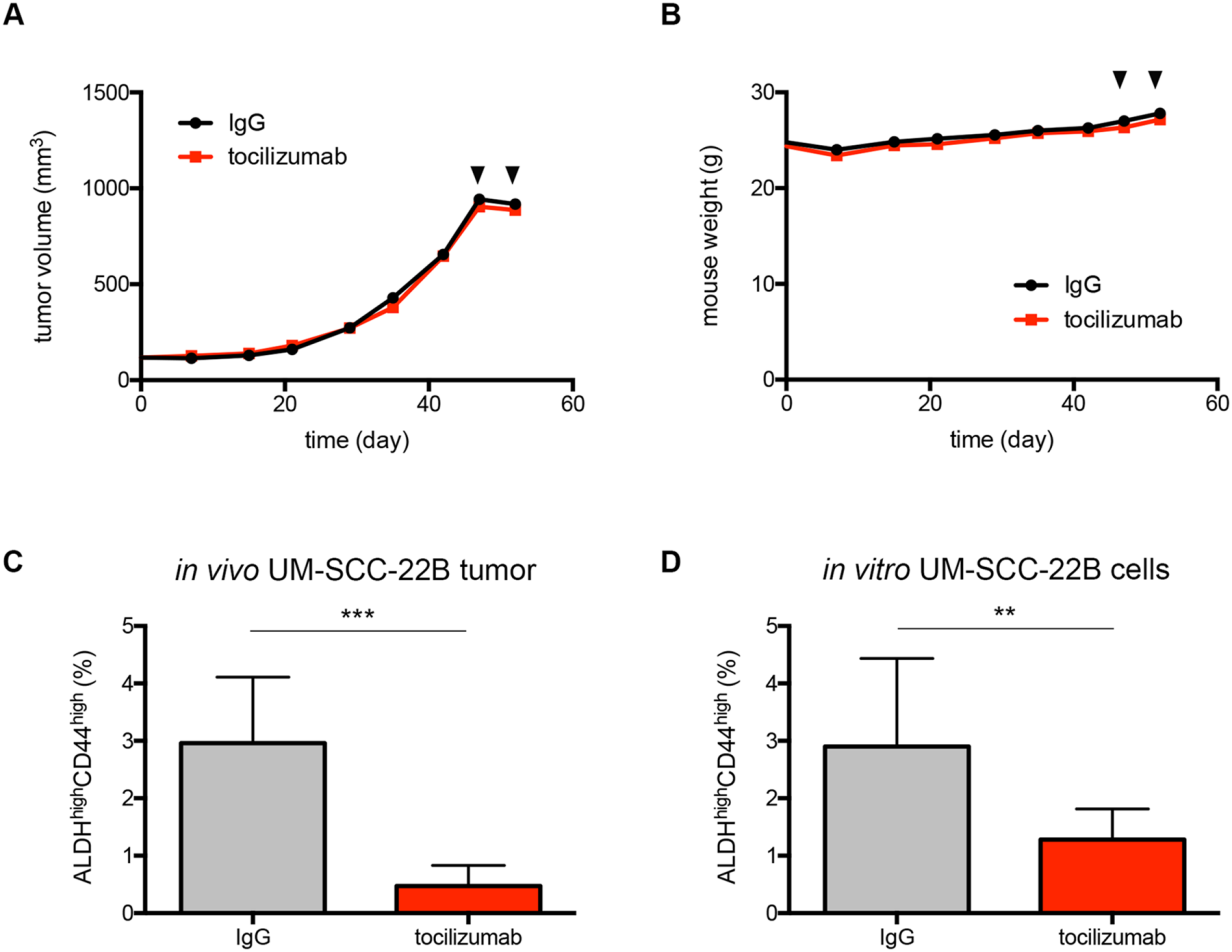
Therapeutic inhibition of the IL-6 pathway decreases the fraction of cancer stem cells **(A)** Graph depicting the tumor volume of xenografts generated upon transplantation of UM-SCC-22B-tumor cells and treated with 2 doses of tocilizumab (arrowheads) (n=12). **(B)** Mouse weight during the study. Arrowheads indicate the two doses of tocilizumab given before tumors were removed. **(C)** Proportion of ALDH^high^CD44^high^ cells in UM-SCC-22B xenograft tumors after tocilizumab treatment detected by FACS analysis. **(D)** FACS analysis result showing the proportion of ALDH^high^CD44^high^ cells in UM-SCC-22B cells after tocilizumab treatment for 24 hours in 10% FBS DMEM *in vitro*. n.s., not significant; ^*^, *P* < 0.05; ^**^, *P* < 0.01; ^***^, *P* < 0.001.

Informed by the TMA results, we assessed the short-term effect of IL-6R inhibition with tocilizumab on the fraction of cancer stem cells in preclinical models of HNSCC. Unsorted UM-SCC-22B cells were co-transplanted with human dermal microvascular endothleial cells (HDMEC) in biodegradable scaffolds to generate xenograft tumors with human vasculature [23], which is amenable to the testing of tocilizumab that does not cross-react with mouse cells [27]. Two doses of tocilizumab (5 kg/mg, IP) were administered within a week period before the tumors were surgically removed. Tocilizumab treatment had no effect on overall tumor volume when compared to IgG treated tumors (Figure 2A). There was no change in mouse weight, indicating that tocilizumab was well tolerated in mice (Figure 2B). FACS analysis of single cell suspensions prepared from the tumor tissues showed a significant decrease in ALDH^high^CD44^high^ cell population in tocilizumab-treated tumors compared to tumors treated with IgG (Figure 2C). In accordance with the *in vivo* data, tocilizumab treatment reduced the ALDH^high^CD44^high^ cell population in UM-SCC-22B cells *in vitro* (Figure 2D). Notably, the concentration of tocilizumab used for *in vitro* experiment did not have cytotoxic effect on tumor cells (Supplementary Figure 2A).

### Endothelial cell-secreted IL-6 supports cancer stem cells and tumor growth

**Figure 3:**
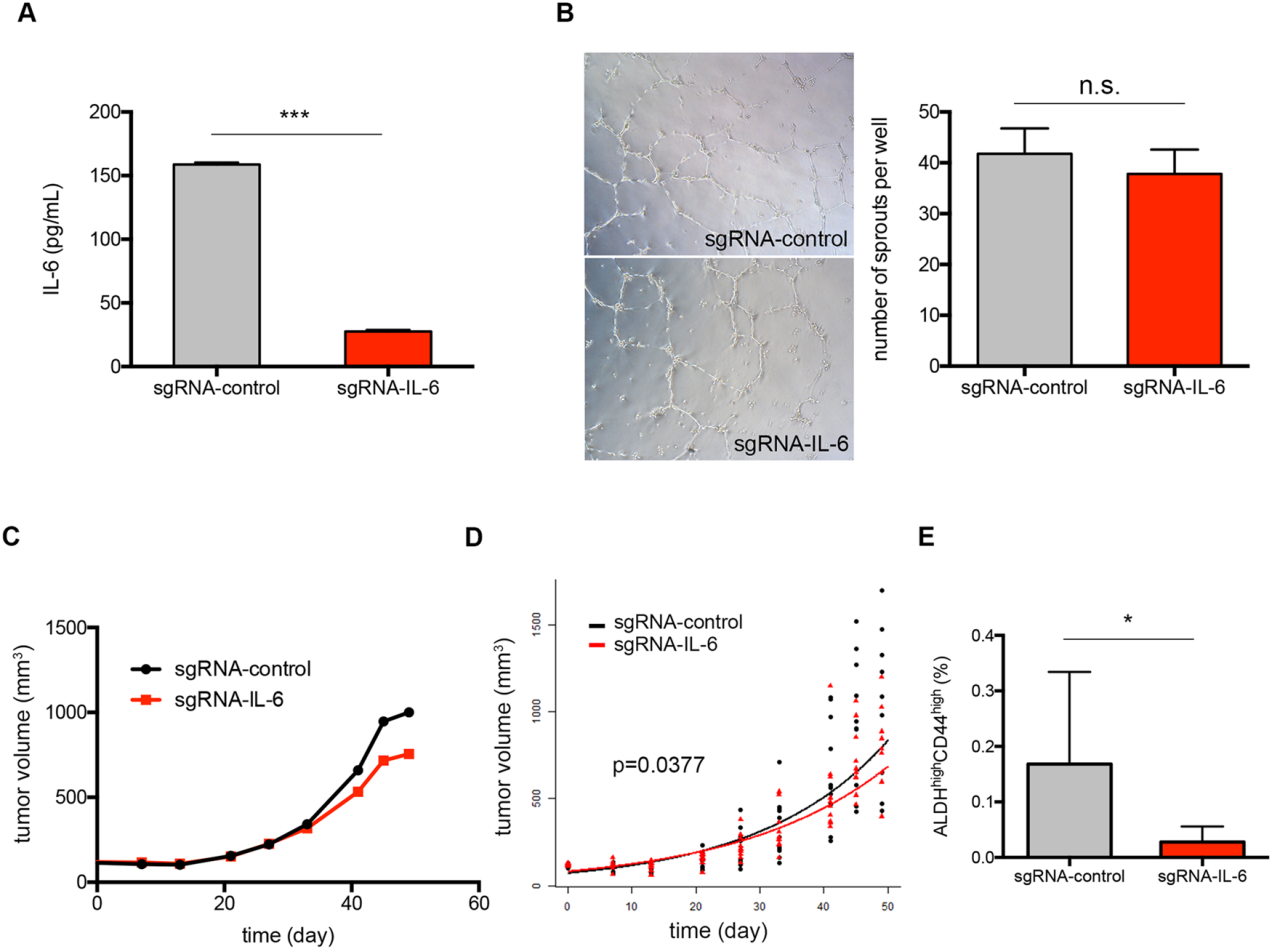
Endothelial cell-secreted IL-6 supports cancer stem cells and tumor growth **(A)** Concentration of IL-6 secreted by sgRNA-control or sgRNA-IL-6 HDMEC quantified by ELISA. **(B)** Representative pictures of sprouts formed by sgRNA-control and sgRNA-IL-6 HDMEC on Matrigel. Average number of sprouts per well is shown as bar graph. **(C)** Graph depicting the average tumor volumes (n=10). **(D)** Repeated measures linear regression estimated mean tumor size prediction line sgRNA-control and sgRNA-IL-6 HDMEC, respectively, overlayed with individual tumor volume. **(E)** FACS analysis of ALDH^high^CD44^high^ cell proportion in tumors grown with sgRNA-control or sgRNA-IL-6 HDMEC. n.s., not significant; ^*^, *P* < 0.05; ^**^, *P* < 0.01; ^***^, *P* < 0.001.

Our group previously reported that head and neck cancer stem cells reside nearby the blood vessels, suggesting functional crosstalk between the two cell types [8]. To test the effect of endothelial cell-secreted IL-6 on the fraction of cancer stem cells *in vivo*, we generated IL-6 knockout endothelial cells (sgRNA-IL-6 HDMEC) using CRISPR/Cas9 system. ELISA assay showed 80% reduction in IL-6 production in sgRNA-IL-6 HDMEC as compared to sgRNA-control HDMEC (Figure 3A). The IL-6 knockout did not affect the vessel forming ability of the endothelial cells, as demonstrated in a Matrigel-based capillary sprouting assay (Figure 3B). sgRNA-IL-6 HDMEC were co-implanted with UM-SCC-22B cells to generate xenograft tumors. Tumor cells grown with sgRNA-IL-6 HDMEC generated smaller tumors compared to tumor cells grown with sgRNA-control HDMEC (Figure 3C). Regression analysis of the tumor growth rates showed that IL-6 knockout in the endothelial cells was sufficient to slow down xenograft tumor growth (Figure 3D). FACS analysis revealed that tumors vascularized with sgRNA-IL-6 HDMEC had a smaller fraction of ALDH^high^CD44^high^ cell population than those vascularized with sgRNA-control HDMEC (Figure 3E).

### Endothelial cell-secreted IL-6 induces cancer stem cell migration

**Figure 4:**
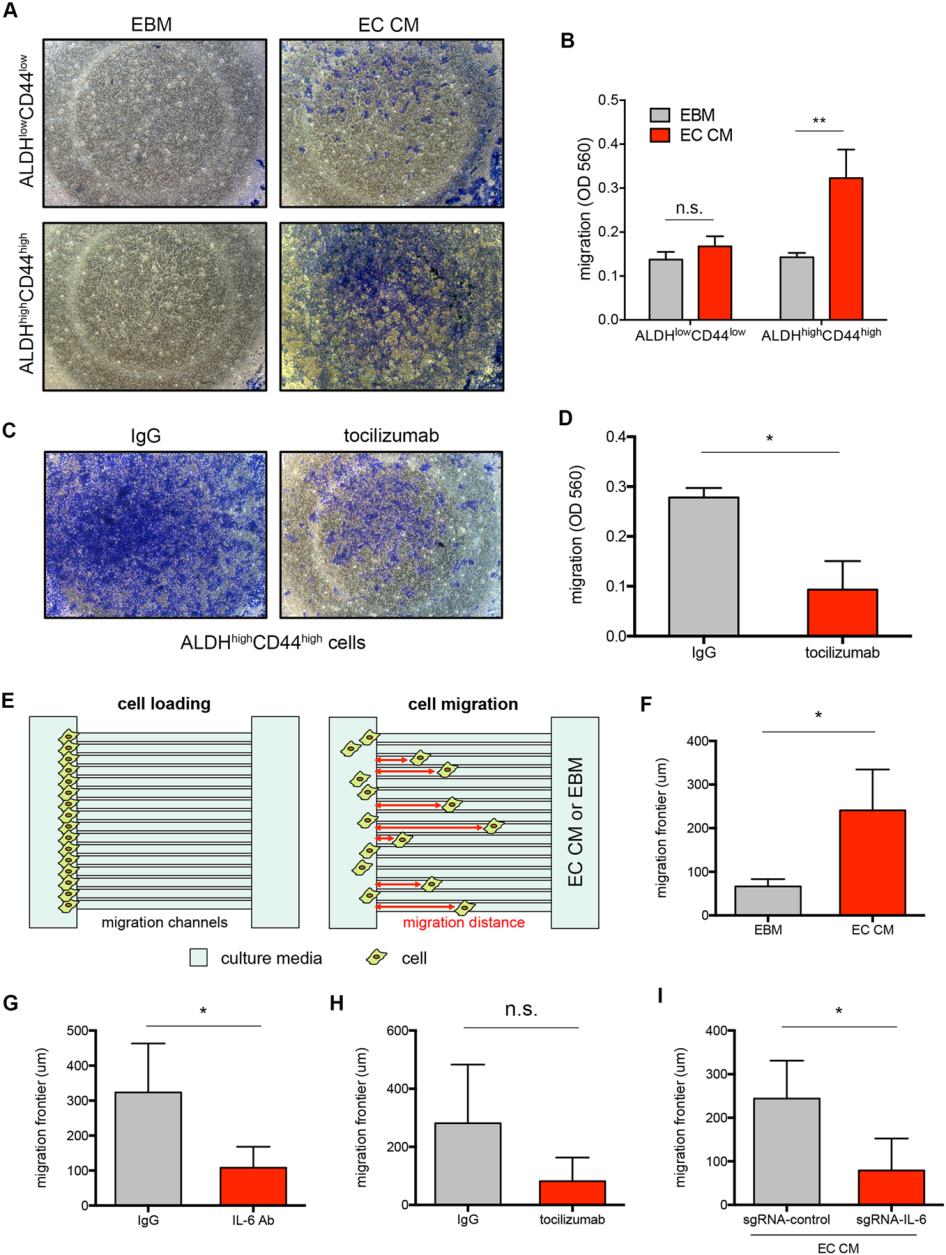
Endothelial cell-secreted IL-6 induces cancer stem cell migration **(A)** Representative pictures of migrated UM-SCC-22B ALDH^low^CD44^low^ or ALDH^high^CD44^high^ cells stained with crystal violet in Transwell insert after 24 hours of incubation in endothelial basal media (EBM) or EC CM. Images taken in 40X magnification. **(B)** Bar graph depicting migrated ALDH^low^CD44^low^ or ALDH^high^CD44^high^ cells over 24 hour-period in Transwell system. **(C)** Representative pictures of ALDH^high^CD44^high^ cells migrated after tocilizumab treatment (2 μg/mL) for 24 hours. Images taken in 40X magnification. **(D)** Quantification of cells migrated after tocilizumab treatment. **(E)** Figure showing the cell loading and migration in microfluidics devices. Migration frontier was calculated by taking the average of individual cell migration distance. **(F)** EC CM induces migration of ALDH^high^CD44^high^ cells in microfluidics device. **(G-I)**, the effect of IL-6 inhibition on ALDH^high^CD44^high^ cells motility. IL-6 signaling was inhibited by neutralizing IL-6 in EC CM (G) treating tumor cells with tocilizumab (H) or using EC CM from sgRNA-IL-6 HDMEC (I). n.s., not significant; ^*^, *P* < 0.05; ^**^, *P* < 0.01; ^***^, *P* < 0.001.

We tested if cancer stem cells had enhanced motility compared to non-cancer stem cells in the presence of endothelial cell conditioned media (EC CM using Transwell migration assay. In the presence of EC CM, more ALDH^high^CD44^high^ cells migrated than ALDH^low^CD44^low^ cells (Figure 4A and 4B). Because the magnitude of migration induction by endothelial cell-secreted factors was stronger in ALDH^high^CD44^high^ cells, we focused on looking at cancer stem cell motility. In order to evaluate the role of endothelial-cell secreted IL-6 on migration of cancer stem cells, we treated sorted ALDH^high^CD44^high^ cells with tocilizumab and allowed the cells to migrate in the presence of EC CM. After 24 hours, we found that tocilizumab reduced the migration of ALDH^high^CD44^high^ cells (Figure 4C and 4D; Supplementary Figure 3A). We repeated the migration experiments using a different approach to verify the reproducibility of the data. Here, we used microfluidics device (Figure 4E; Supplementary Figure 3B and Supplementary Video) that was previously described [24, 25]. EC CM induced strong migration of ALDH^high^CD44^high^ cells (Figure 4F; Supplementary Figure 3C). We observed a reduction in cancer stem cell migration when the IL-6 pathway was inhibited either with an IL-6 neutralizing antibody (Figure 4G) or with tocilizumab (Figure 4H). To validate the data obtained with antibodies target to the IL-6 pathway, we performed migration studies using as chemotactic stimulus the EC CM from sgRNA-IL-6 HDMEC. Again, migration of ALDH^high^CD44^high^ cells was reduced (Figure 4I; Supplementary Figure 3D).

### Endothelial cell-secreted IL-6 induces expression of mesenchymal markers in head and neck cancer stem cells

**Figure 5:**
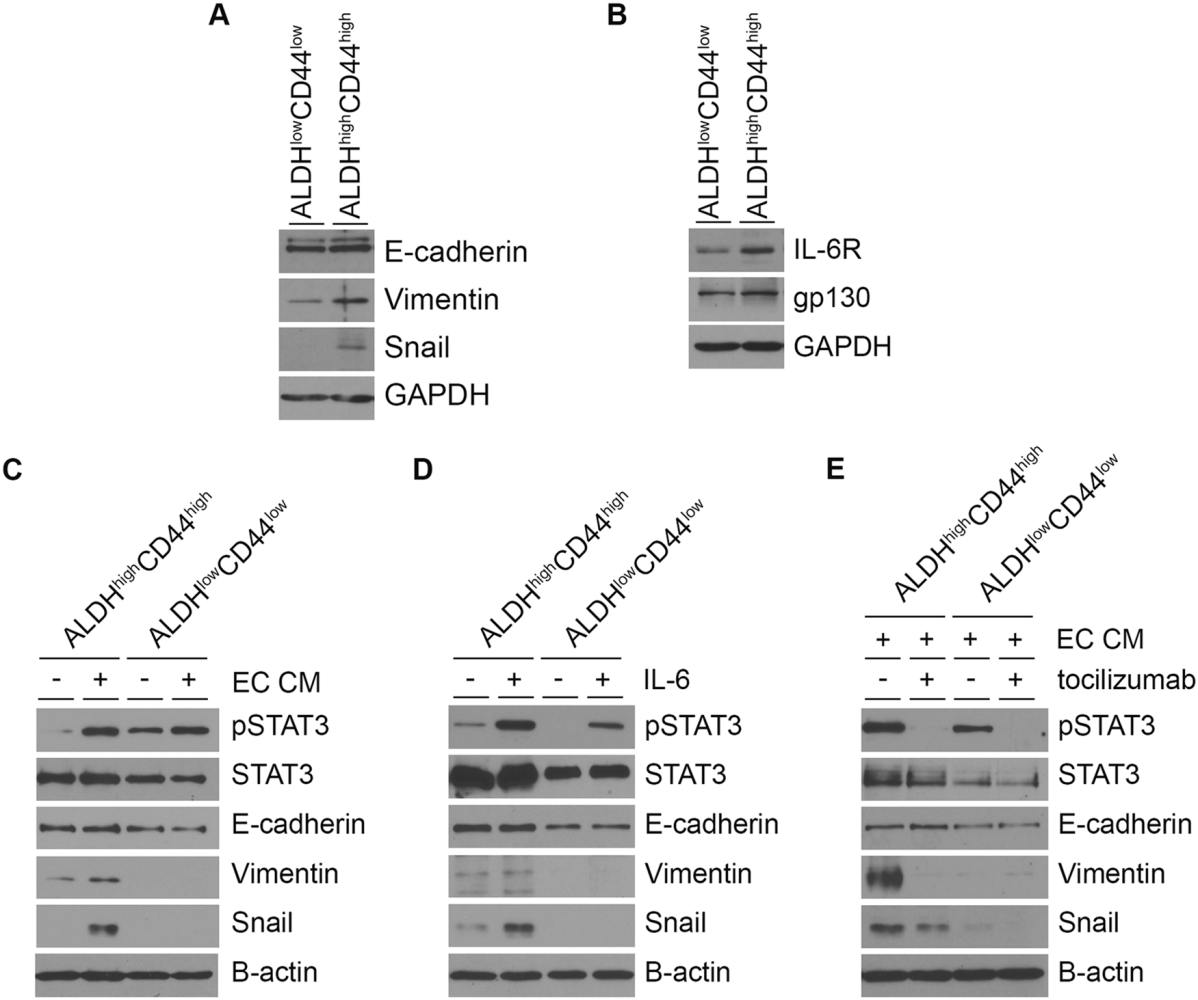
Endothelial cell-secreted IL-6 induces mesenchymal cell marker expression in head and neck cancer stem cells **(A)** EMT state of UM-SCC-22B ALDH^high^CD44^high^ and ALDH^low^CD44^low^ cells. **(B)** IL-6R and gp130 levels in ALDH^high^CD44^high^ cells compared to ALDH^low^CD44^low^ cells. **(C), (D), (E)** Effect of EC CM (C), IL-6 (D) and tocilizumab (E) on EMT markers in ALDH^high^CD44^high^ cells and ALDH^low^CD44^low^ cells.

The results from migration experiments led to our speculation that the enhanced migratory phenotype of cancer stem cells might be associated with induction of EMT. Indeed, ALDH^high^CD44^high^ cells expressed higher levels of mesenchymal cell-related proteins, Vimentin and Snail, as compared with ALDH^low^CD44^low^ cells (Figure 5A). Interestingly, we found that ALDH^high^CD44^high^ cells expressed higher levels of IL-6R and its co-receptor gp130 than ALDH^low^CD44^low^ cells (Figure 5B). Then, we tested whether the enhanced migratory ability of ALDH^high^CD44^high^ cells in the presence of EC CM correlates with differential EMT level. In order to maintain cancer stem cell population after sorting, sorted tumor cells were grown in spheres. We observed that EC CM induced Vimentin and Snail expression in ALDH^high^CD44^high^ cells but not in ALDH^low^CD44^low^ cells (Figure 5C; Supplementary Figure 4B). Similar results were reproduced in ALDH^high^CD44^high^ cells treated with recombinant IL-6 (Figure 5D). Notably, IL-6R blockade with tocilizumab inhibited endothelial cell-induced Vimentin and Snail expression in ALDH^high^CD44^high^ cells (Figure 5E; Supplementary Figure 4C). We did not observe any change in E-cadherin expression in both ALDH^high^CD44^high^ and ALDH^low^CD44^low^ cells (Figure 5C-5E). This might be because E-cadherin is essential in cells surviving in anchorage-independent environment [28].

### STAT3 regulates mesenchymal cell markers in head and neck cancer stem cells

**Figure 6:**
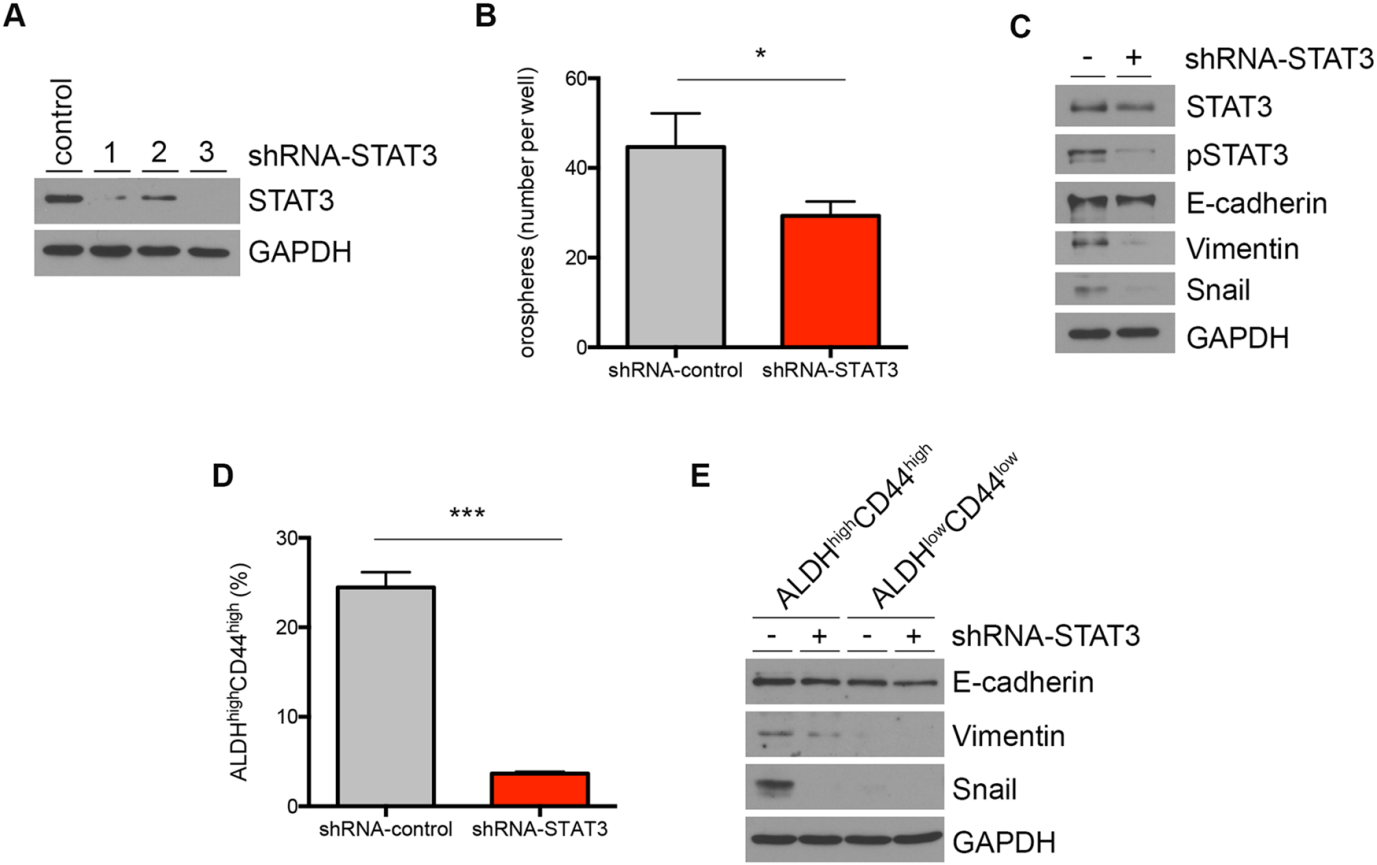
STAT3 regulates mesenchymal cell markers in head and neck cancer stem cells **(A)** Western blot showing STAT3 knockdown efficiencies of different constructs. The construct #3 was used for the experiments depicted in panels B-E. **(B)** Bar graph depicts average number of orospheres generated from shRNA-control or shRNA-STAT3 UM-SCC-22B. **(C)** Western blot of STAT3 knockdown cells probing for STAT3, pSTAT3, E-cadherin, Vimentin, Snail and GAPDH. **(D)** FACS analysis of ALDH^high^CD44^high^ cell percentage in STAT3 knockdown UM-SCC-22B cells. **(E)** E-cadherin, Vimentin, Snail and GAPDH detection in ALDH^high^CD44^high^ cells with STAT3 knockdown. n.s., not significant; ^*^, *P* < 0.05; ^**^, *P* < 0.01; ^***^, *P* < 0.001.

It is well known that STAT3 is a key downstream effector of IL-6 signaling through IL-6R [29]. We observed that STAT3 was phosphorylated in ALDH^high^CD44^high^ cells treated with EC CM or recombinant human IL-6, and that tocilizumab inhibited STAT3 phosphorylation (Figure 5C-5E). Here, we silenced STAT3 in tumor cells using shRNA constructs (Figure 6A; Supplementary Figure 5A). STAT3 knockdown resulted in decreased orosphere-forming ability of HNSCC cells in ultralow attachment plates, suggesting the importance of STAT3 signaling to the survival and self-renewal of head and neck cancer stem cells (Figure 6B; Supplementary Figure 5B). We tested the role of STAT3 on mesenchymal cell-related protein expression in HNSCC cells. STAT3 silencing of unsorted HNSCC cells was associated with lower Vimentin and Snail expressions (Figure 6C). Further, STAT3 silencing resulted in a significant decrease in the ALDH^high^CD44^high^ cell population (Figure 6D; Supplementary Figure 5C). Notably, STAT3 silencing inhibited expression of the mesenchymal cell-related markers, Snail and Vimentin, in ALDH^high^CD44^high^ cells (Figure 6E).

## DISCUSSION

Tumor dissemination is the major problem in the management of patients with advanced head and neck cancer. Greater than half of patients with locally advanced head and neck cancer develop tumor metastasis or relapse, and the survival rate for patients with metastasis or relapse is between 5 to 9 months [30]. However, our understanding of the mechanisms leading to metastatic spread is still limited. It has been recently proposed that cancer stem cells play a critical role in metastasis development in several cancer types [31–33]. Chinn et al. highlighted enhanced potential of cancer stem cells to generate lymph node metastasis in HNSCC [34]. Although the importance of cancer stem cells in the metastatic process is well recognized, it is still unclear how cancer stem cells move away from the tumor nests towards blood vessels. Here, we demonstrated endothelial cell-secreted IL-6 induces cancer stem cell migration and also unveiled the therapeutic potential of IL-6 signaling blockage in HNSCC.

The importance of IL-6 on cancer pathobiology has been well recognized from different types of tumors. Serum level IL-6 has been identified as prognostic marker in many types of cancer, including ovarian cancer [35], prostate cancer [36], breast cancer [14], colon cancer [37], melanoma [38] and HNSCC [16]. Here, we observed that high IL-6R or gp130 level in the invasive front of tumors correlated with poor outcome in HNSCC patients. IL-6 signaling through IL-6R/gp130 induces robust activation of STAT3 signaling. It has been shown that STAT3 activation enhances invasion and motility of tumor cells [39], contributing to the aggressiveness of the tumor. To our knowledge, this work is the first to demonstrate that high IL-6R or gp130 in the invasive front of the tumor are independent predictive markers of HNSCC patient overall survival.

With mounting evidence showing the importance of IL-6 in tumor biology, IL-6 pathway targeting drugs that either block the receptor or binding the ligand have been tested. Tocilizumab is a humanized anti-IL-6R antibody that inhibits both soluble and membrane-bound IL-6R to prevent IL-6 pathway activation. The half-life of the antibody is long enough (11-13 days depending on the concentration) to allow monthly intravenous injections [40]. The drug is well tolerated by the patients with minimal side effects [40]. In this study, we tested the effect of tocilizumab on HNSCC tumors and found that two doses of tocilizumab are sufficient to reduce the cancer stem cell fraction. Notably, we performed a short-term experiment to single out the effect of IL-6R inhibition with tocilizumab on the cancer stem cells before the therapy caused major differences in overall tumor size which would introduce other confounders (*e.g.* larger tumors may contain more necrotic cores, increased inflammatory infiltrates...). We have also previously reported that inhibition of IL-6R with tocilizumab, or direct inhibition of IL-6 with a neutralizing antibody, inhibits orosphere formation with HNSCC cells [21, 41]. Such results highlight the importance of IL-6 pathway in the survival of head and neck cancer stem cells and the therapeutic potential of IL-6 inhibition in HNSCC. We have previously shown that cisplatin increases the cancer stem cell fraction in pre-clinical model of HNSCC [42], suggesting a possible explanation for why conventional therapies fail to prevent metastasis and recurrence of head and neck cancer. The combination of chemotherapy and tocilizumab may reduce the tumor size (*i.e.* debulk the tumor) while at the same time ablate the cancer stem cells and reduce the chances of tumors to metastasize or relapse.

Our group recently reported that treatment of tocilizumab alone slowed the growth rate of xenograft model of mucoepidermoid carcinoma tumors, another subtype of head and neck cancer [43]. Importantly, the tumor growth rate and the fraction of cancer stem cells significantly decreased when the tumors were treated with tocilizumab and cisplatin together, suggesting that combination of existing chemotherapy and IL-6 pathway blocking agent may also be beneficial for other head and neck cancer patients.

Here, we observed that IL-6 knockout endothelial cells resulted in slow tumor growth with smaller fraction of cancer stem cells. These results suggest that endothelial cells are a key source of the IL-6 that is required to maintain the cancer stem cell population in the perivascular niche, which perhaps modulates the aggressiveness of the tumor. It has been shown that IL-6 induces EMT in breast cancer models [44], and that it promotes metastasis to lymph node and lungs in HNSCC [17]. Indeed, we and others reported that inhibition of the IL-6 pathway inhibits migration of HNSCC [17, 45]. Further, we showed that head and neck cancer stem cells express more mesenchymal cell-related proteins and are more motile than non-cancer stem cells in response to endothelial cell-secreted factors. We demonstrated that inhibition of endothelial cell-activated IL-6 pathway prevented EMT in cancer stem cells and inhibited their migration, unveiling the role of IL-6 on cancer stem cell motility. This was observed even in presence of the IL-6 secreted by the head and neck tumor cells, which we know is at lower constitutive levels than the IL-6 expressed by the endothelial cells [21]. Interestingly, we did not observe induction of migration upon recombinant human IL-6 treatment alone (data not shown). By itself, IL-6 might endow cancer stem cells with migratory phenotype via EMT without necessarily acting as chemokine to induce cell movement. In contrast, the full endothelial cell-secreted milieu is very efficient at inducing cancer stem cells migration, and within this milieu IL-6 plays a critical role as demonstrated in several blocking experiments. We conclude that tumor cell migration towards blood vessels might be the result of endothelial cell-secreted factors working together, and that IL-6 primes the cancer stem cells to respond to these factors. We postulate that tumor cells may acquire migratory phenotype through the IL-6/IL-6R/STAT3 axis, and potent chemokines, such as CXCL8, initiate the actual cellular movement to a specific direction.

A major challenge in studying cancer stem cells is the rarity of this cell population. Head and neck cancer stem cell proportion ranges between 0.6-4.5% in primary tumors [46] and 1-10% in cell lines [11]. Working with such small population of cells makes it difficult to do in-depth analysis with most common research tools, as the unique responses of cancer stem cells may not be reflected in studies with bulk cells. Recent advances in microfluidics technologies have enabled cancer biologists to answer questions that were once thought not possible to answer. For example, microfluidics devices allowed isolating circulating tumor cells from blood samples [47] and looking at RNA levels of single cells after different treatment [48]. Our group used a novel microfluidics migration platform to assess the migration ability of cancer stem cells in the presence of EC CM and showed the impact of IL-6 pathway in cancer stem cell motility. Indeed, the results seen from microfluidic devices were in parallel with Transwell migration assays performed with sorted cancer stem cells. In comparison to Transwell system, microfluidics platform creates gradient of EC CM within the migration channel, thus emulating the biological environment more accurately. In addition, the microfluidics devices allow analysis of the movement of single cells during migration process.

**Figure 7:**
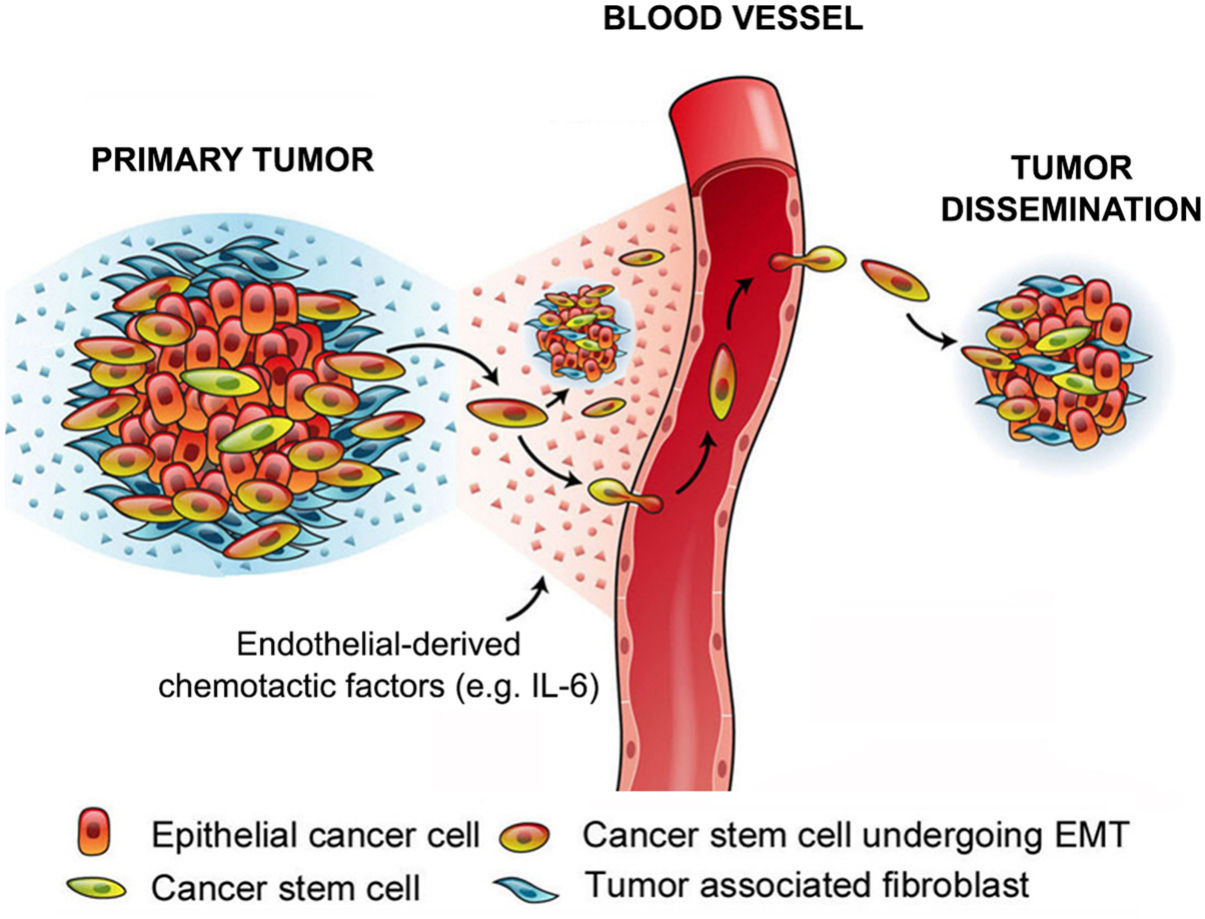
Diagram depicting the overall premise of this work We showed here that endothelial cell-derived IL-6 induces EMT and generates a chemotactic gradient for cancer stem cells enabling their movement towards vascular endothelial cells. We postulate that this process constitutes the initial step for metastatic dissemination of epithelial tumors, and propose the development of a new mechanism-based therapy that is based on the combination of short-term chemotherapy with long-term IL-6 blockade for prevention of tumor recurrence/metastasis.

In conclusion, we demonstrated here the impact of endothelial cell-initiated IL-6 signaling to the migratory phenotype of head and neck cancer stem cells, which are (Figure 7) the primary mediators of HNSCC tumor dissemination. These results suggest the possibility that a combination therapy involving conventional chemotherapy to debulk the majority of the more differentiated tumor cells together with an IL-6 pathway-inhibiting agent to ablate cancer stem cells might be beneficial for patients with HNSCC.

## MATERIALS AND METHODS

### Cell culture and reagents

HNSCC cell lines UM-SCC-1 and UM-SCC-22B (Tissue Biorepository, University of Michigan Head and Neck SPORE) were cultured in Dulbecco’s Modified Eagle’s Medium (DMEM; Invitrogen, Carlsbad, CA, USA) with 10% FBS, 100 U/mL penicillin, and 100 U/mL streptomycin (Invitrogen). Primary human dermal microvascular endothelial cells (HDMEC; Lonza, Walkersville, MD, USA) were cultured in endothelial growth medium-2 for microvascular cells (EGM2-MV; Lonza). Endothelial cell conditioned medium (EC CM) was prepared by collecting supernatant from 24-hour culture in serum-free endothelial basal medium (EBM2; Lonza). IL-6 signaling pathway was inhibited by treating cells with 2 μg/mL tocilizumab (Genentech, South San Francisco, CA, USA) or EC CM with 1 μg/mL anti-IL-6 neutralizing antibody (R&D Systems, Minneapolis, MN, USA).

### Tissue microarray, immunohistochemistry and immunofluorescence

The preparation of the tissue microarray (TMA) slides is described elsewhere [22]. Tissue sections were deparaffinized in xylene, washed with 100% ethanol and rehydrated with graded ethanol. Antigen retrieval was performed by boiling the slides in 1X citrate buffer (Thermo Scientific, Fremont, CA, USA) for 20 minutes. After slides were cooled down to room temperature, the TMA slides were permeabilized for 10 minutes at room temperature, and endogenous peroxidase activity was inhibited by 10-minute incubation with 3% hydrogen peroxide (Fisher, Waltham, MA, USA). Mouse monoclonal anti-human gp130 (1:100 dilution; Santa Cruz) and IL-6Rα (1:100 dilution; Abcam) were incubated overnight at 4°C. Chromogenic development was achieved by incubating DAB peroxidase substrate (Biocare Medical, Concord, CA, USA) for 1-5 minutes at room temperature. Two pathologists blind to patient information scored the stained sections based on the staining intensity (1=no staining, 2=moderate staining, 3=intense staining) and percentage of positive cells (0=0-10%, 1=10-50%, 2=greater than 50%). Final score was calculated by multiplying the intensity and positive scores. Patients were divided to low (score ≤4) and high (score >4) groups. Same deparaffinization and antigen retrieval steps were performed for immunofluorescence assay. Primary antibodies, ALDH1A1 (1:100 dilution; BD Biosciences) and IL-6Rα (1:100 dilution; Santa Cruz), were incubated in 4°C overnight. Sections were washed with PBS (Invitrogen) and incubated in fluorochrome-conjugated secondary antibodies for 20 minutes at room temperature. After another PBS wash, the slide was mounted with DAPI mounting solution and covered with coverslip. Nikon Eclipse 80i fluorescence microscope was used to take immunofluorescence images, and NIH ImageJ was used to process images.

### Effect of IL-6R inhibition on tumor growth

1-2x10^5^ UM-SCC-22B cells and 8-9x10^5^ HDMEC were seeded in a biodegradable scaffold, as we described (23). Loaded scaffold was implanted bilaterally in subcutaneous space of the dorsal region of severe combined immunodeficient mouse (CB.17.SCID; Charles River, Wilmington, MA, USA). Tumors were measured weekly, and tumor volume was calculated using the formula, (width x width x length)/2. Tocilizumab (5 mg/kg) or control IgG was given via intraperitoneal injection. Mouse weight was measured weekly to observe any adverse effect from the treatment.

### Fluorescence activated cell sorting (FACS) analysis

Head and neck cancer stem cells were isolated as described previously [8]. Briefly, single cell suspension was prepared from cell culture and resuspended at 1x10^6^ cells/mL. 5 μL activated Aldefluor (BODIPY-aminoacetate) (Aldefluor kit; Stem Cell Technologies, Vancouver, BC, Canada) or negative control (DEAB; diethylaminobenzaldehyde) was added and incubated at 37°C for 35 minutes. Then the cells were washed and incubated with 5 μL anti-human CD44 (BD Pharmingen, Pharmingen, NJ, USA) at 4°C for 30 minutes. 5 μL 7-aminoactinomycin (BD Pharmingen) was used to select out dead cells. Head and neck cancer stem cells were defined as ALDH^high^CD44^high^ and non-cancer stem cells as ALDH^low^CD44^low^.

### Migration assay

Transwell migration assay was performed on HTS Transwell 96 well permeable support (Corning, Corning, NY, USA). Inserts were coated with 0.2% gelatin (Sigma-Aldrich, St. Louis, MO, USA) for 15 minutes at room temperature prior to cell loading. 5x10^4^ sorted ALDH^high^CD44^high^ or ALDH^low^CD44^low^ cells resuspended in 50 μL DMEM were loaded to each insert. 200 μL of EC CM was loaded at the bottom well. Transwell plate was incubated in cell culture incubator for 24 hours. Cells that did not migrate were scrapped off from the top of the insert. Migrated cells were stained with 0.5% crystal violet (Sigma-Aldrich) solution in 25% methanol (Fisher Scientific, Fair Lawn, NJ, USA) for 20 minutes at room temperature. Migration was quantified by dissolving the crystal violet staining in 10% acetic acid (Fisher Scientific) and reading absorbance at 560 nm. Alternatively, cell migration assays were performed using a microfluidics migration platform that we previously developed [24, 25]. Prior to cell loading, the device was coated with 0.1 mg/mL collagen solution (rat tail collagen type 1; BD Biosciences) for 1 hour to enhance cell adhesion and viability. The device was rinsed with PBS (Invitrogen) for 1 hour to remove the residual collagen. Sorted ALDH^high^CD44^high^ or ALDH^low^CD44^low^ cells were resuspended to 3x10^5^ cell/mL concentration for loading. After cell loading, the cell suspension in the left inlet was replaced with serum-free DMEM, and EC CM was applied to the other inlet to induce migration. Migration frontier was measured by taking the average of distances the cells migrated after 24 hours of incubation without media replenishment.

### Generation of IL-6 knockout endothelial cells

IL-6 knockout (sgRNA-IL-6) HDMEC cells were generated using CRISPR/Cas9 system [26]. lentiCRISPR v2 was a gift from Feng Zhang (Addgene plasmid # 52961). HEK293T cells were transfected with cocktail of pMD2G, psPAX2, and lentiCRISPR v2 with IL-6 guide sequences (5’-GGTCCAGTTGCCTTCTCCCT-3’ and 5’-GTTCCTGCAGTCCAGCCTGA-3’) using calcium phosphate method. HDMEC were incubated in HEK293T supernatant with 4 μg/mL polybrene (Sigma-Aldrich) overnight and maintained in 1 μg/mL puromycin (InvivoGen, San Diego, CA, USA) EGM2-MV for 2 weeks. IL-6 knockout efficiency was evaluated by performing ELISA (R&D Systems) assay with sgRNA-IL-6 EC CM.

### Generation of STAT3 knockdown HNSCC cells

The calcium phosphate method was used to transfect shRNA-control (scramble control) or shRNA-STAT3 constructs (University of Michigan Vector Core) with pMD2G and psPAX2 package vectors into HEK 293T cells. HNSCC cells were infected with transfected HEK293T supernatant overnight with 4 μg/mL polybrene (Sigma-Aldrich). Infected tumor cells were cultured in 1 μg/mL puromycin (InvivoGen) added DMEM medium for 2 weeks.

### Western blot

Sorted ALDH^high^CD44^high^ and ALDH^low^CD44^low^ cells were plated in 6-well ultra-low attachment plate (Corning) and incubated for 24 hours. Protein lysates were prepared using 1% Nonidet P-40 (NP-40) buffer. 10-20 μg protein was loaded in 9-11% SDS-PAGE gel. Primary antibodies include: phosphorylated-STAT3 (1:1000 dilution), STAT3 (1:10,000 dilution), Vimentin (1:500 dilution) and Snail (1:1000 dilution) from Cell Signaling (Danvers, MA, USA); E-cadherin (1:1000 dilution) and β-actin (1:10000 dilution) antibody from Santa Cruz; GAPDH (1:10000 dilution) antibody from Chemicon (Temecula, CA).

### Sprouting assay

5X10^4^ HDMEC per well were plated in 12-well plates (BD Falcon, NJ, USA) coated with growth factor-reduced Matrigel (Corning). After 24 hour incubation in 37°C 5% CO_2_, the number of capillary-like sprouts was counted to determine the impact of IL-6 knockout on *in vitro* angiogenic potential.

### Statistical analysis

Tumor microarray survival time data was analyzed using log-rank test or multivariate Cox proportional hazards models. Tumor volume growth rate was assessed using linear mixed effect models to account for repeated measurements with an auto-regressive correlation structure assuming more correlation among temporally proximate observations. The tumor size was log transformed to account for exponential tumor volume growth. Model fixed effects included time and IL-6 knockout status, and model random effects included tumor. Survival analysis was performed using the “survival” package and mixed effect regression was performed using the “nlme” package, both in the statistical software program R version 3.1.0. Unpaired t test was used to determine significance. *P* ≤ 0.05 was considered significant (n.s., not significant; ^*^, *P* ≤ 0.05; ^**^, *P* ≤ 0.01; ^***^, *P* ≤ 0.001). Comparisons in means were performed using Prism software (GraphPad Software, La Jolla, CA, USA).

## SUPPLEMENTARY MATERIALS FIGURES AND VIDEO





## Abbreviations

HNSCC: head and neck squamous cell carcinoma
IL-6: interleukin-6
IL-6R: interleukin-6 receptor
ALDH: aldehyde dehydrogenase
EMT: epithelial-mesenchymal transition
EC: endothelial cells
CM: conditioned medium
