# Supplementary material for: Endothelial-derived interleukin-6 induces cancer stem cell motility by generating a chemotactic gradient towards blood vessels
Source: Oncotarget. 2017 Nov 1;8(59):100339–52. doi: 10.18632/oncotarget.22225 (PMC5725024; doi:10.18632/oncotarget.22225)
Supplement: Supplementary file 1 [file oncotarget-08-100339-s001.pdf]

Endothelial-derived interleukin-6 induces cancer stem cell motility by generating a chemotactic gradient towards blood vessels

SUPPLEMENTARY MATERIALS

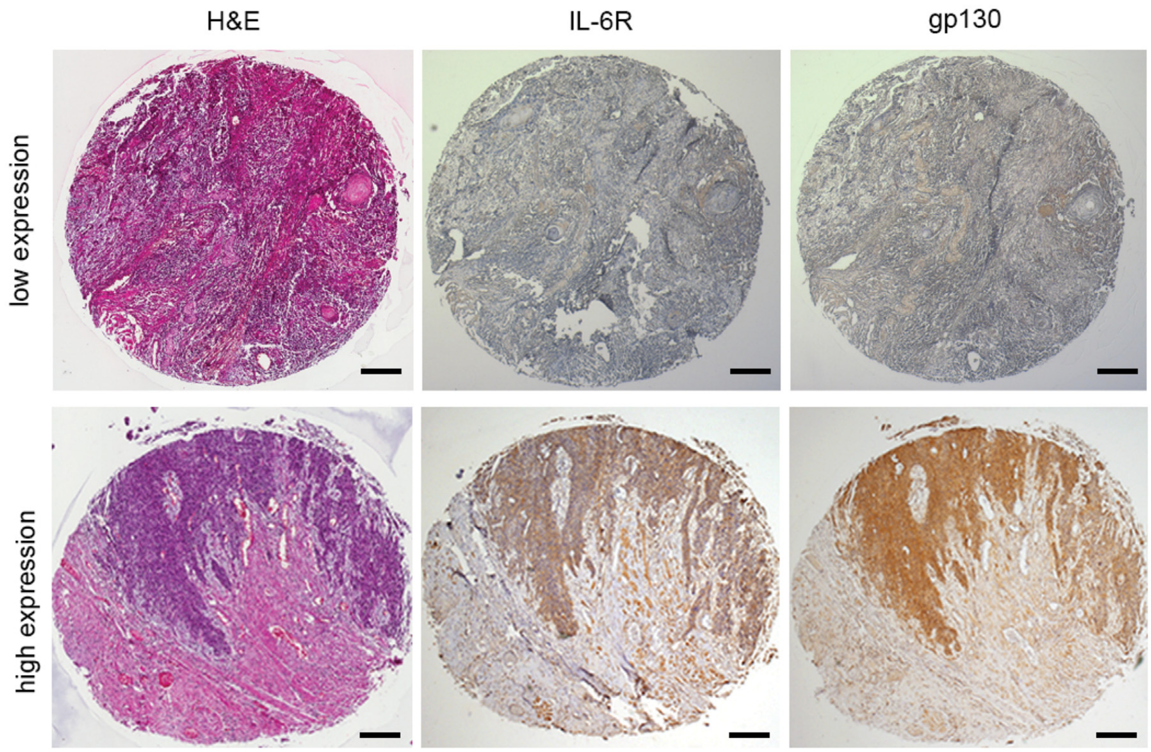

Supplementary Figure 1: Representative images of tissue microarray (TMA) immunostaining of IL-6R and gp130. Please note that the tissue cores used to build this TMA were retrieved from the invasive fronts of head and neck squamous cell carcinomas. Scale bars = 250 µm.

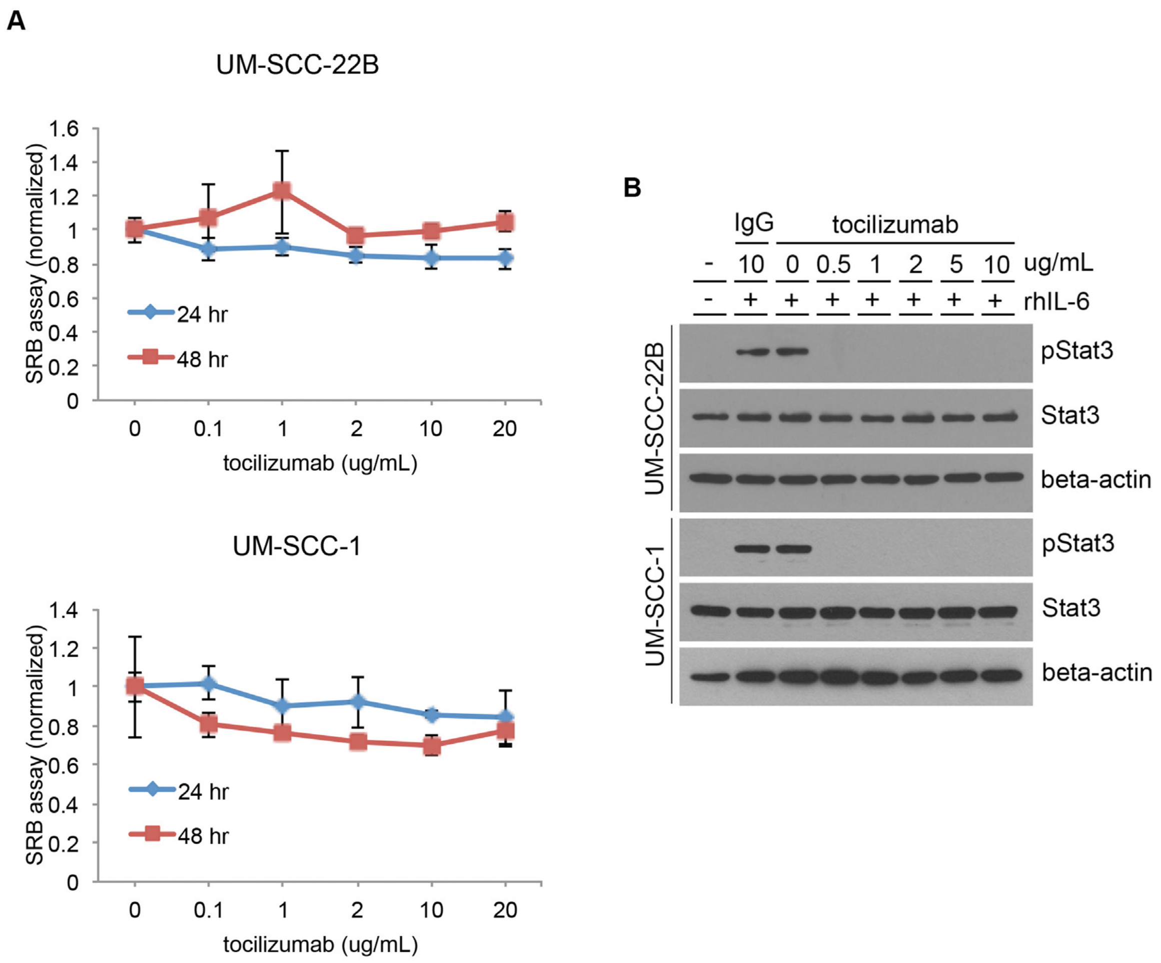

Supplementary Figure 2: Tocilizumab concentration used for the experiment did not affect the cell viability. (A) Sulforhodamine, **(B)** (SRB) assay result of UM-SCC-22B and UM-SCC-1 after 24- and 48-hour tocilizumab treatments. (B) Western blot of UM-SCC-22B and UM-SCC-1 cells treated with different concentrations of tocilizumab to inhibit phosphorylation of STAT3. We used 2 µg/mL tocilizumab for all *in vitro* assays.

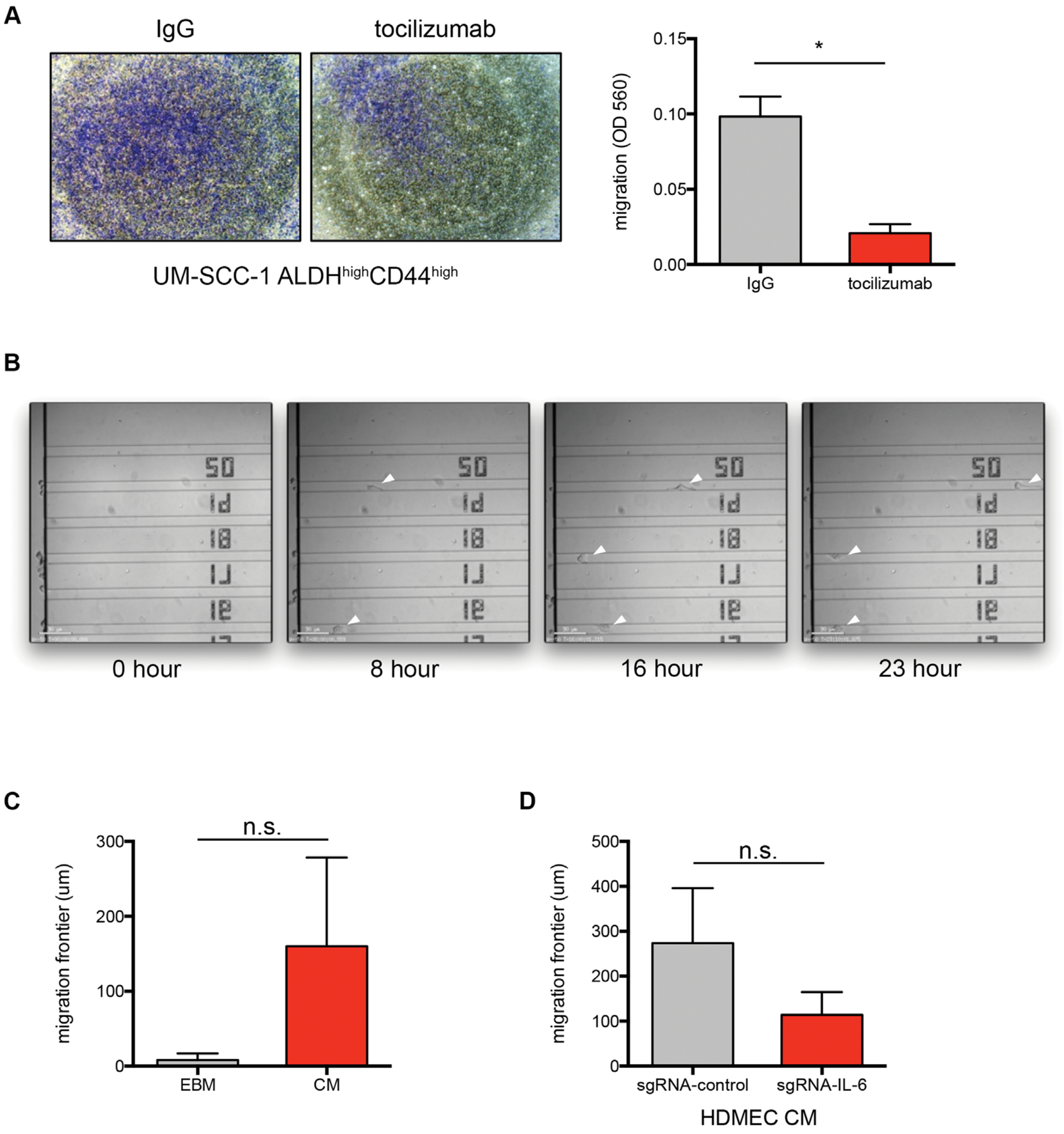

Supplementary Figure 3: Blockage of endothelial cell-initiated IL-6 pathway reduces migration of UM-SCC-1 cancer stem cells. **(A)** Representative pictures of migrated cells in transwell 24 hours after tocilizumab treatment. Pictures are taken in 40X magnification. Bar graph depicts the OD560 level of crystal violet stained migrated cells. **(B)** Time-lapse images of tumor cells migrating through the migration channel of microfluidics device over 24 hour period in the presence of endothelial cell CM. **(C & D)** Migration frontiers of ALDH^{high}CD44^{high} cells in the presence of EC CM or sgRNA-IL-6 HDMEC CM. n.s., not significant; *, $P \leq 0.05$; **, $P \leq 0.01$; ***, $P \leq 0.001$.

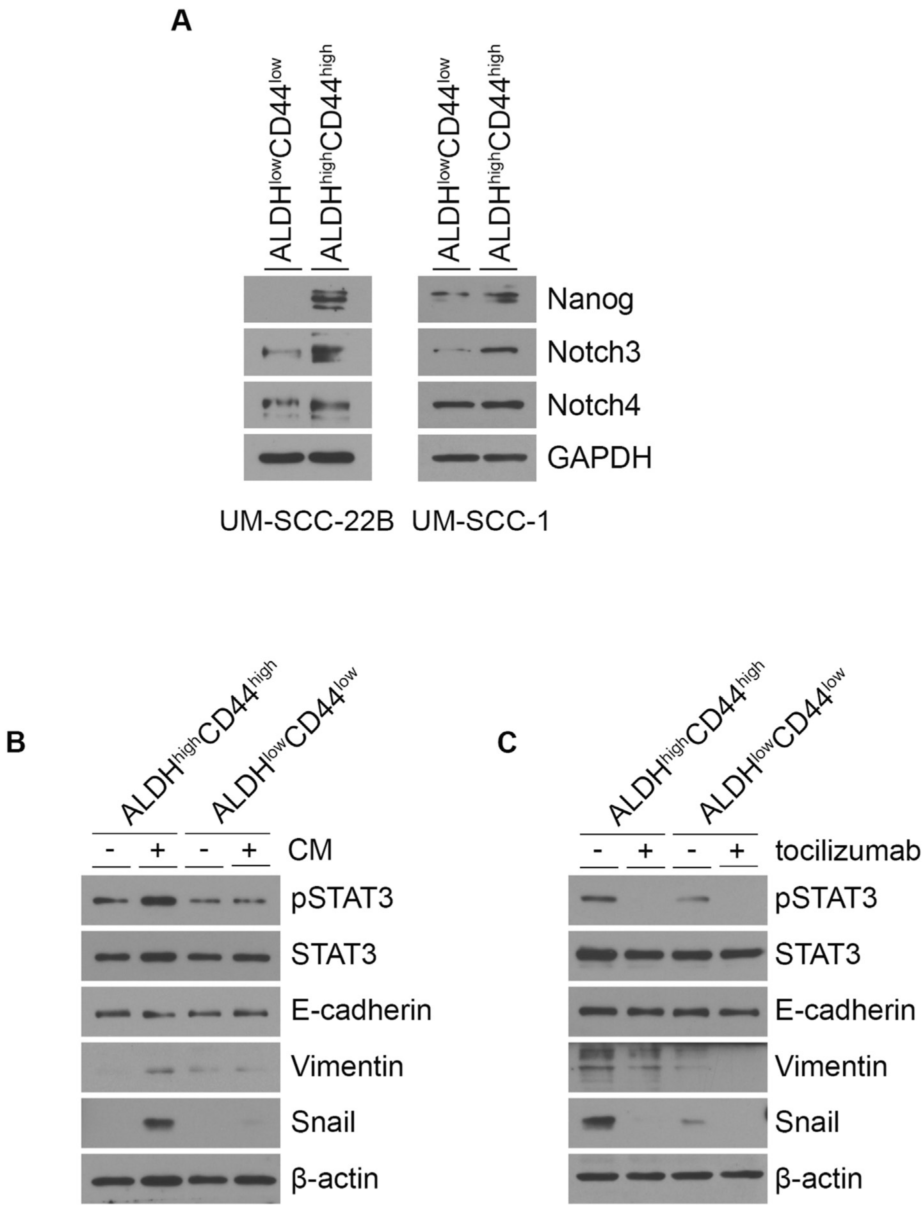

Supplementary Figure 4: Endothelial cell-secreted IL-6 increases stemness and induces EMT in head and neck cancer stem cells. (A) Stemness-related protein expressions in ALDH^{high}CD44^{high} and ALDH^{low}CD44^{low} cells in UM-SCC-22B and UM-SCC-1. B, EMT protein markers expressed in UM-SCC-1 ALDH^{high}CD44^{high} and ALDH^{low}CD44^{low} cells with EC CM **(B)** and tocilizumab treatment **(C)**.

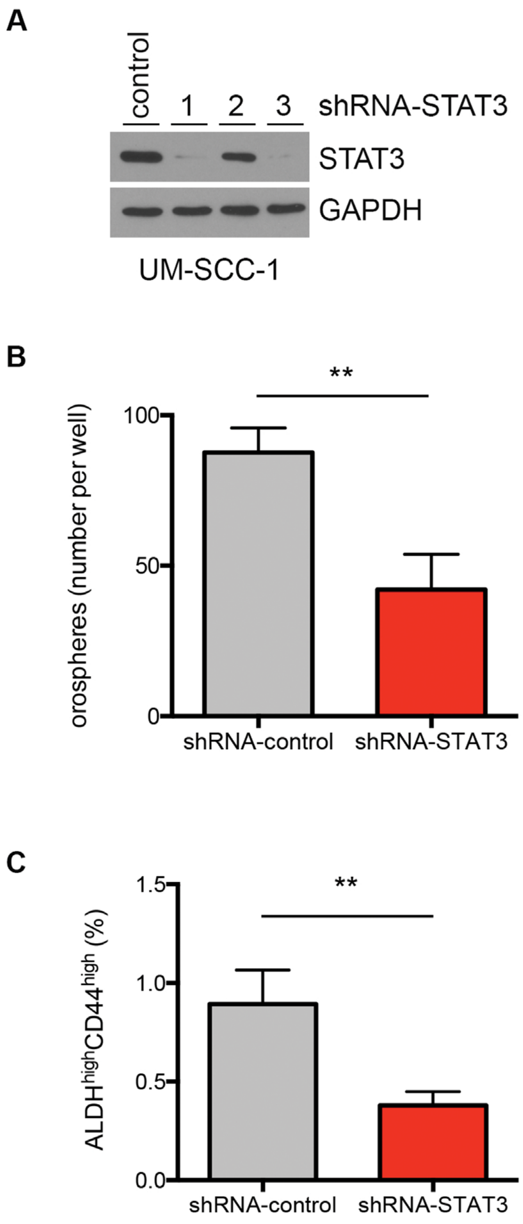

Supplementary Figure 5: Effect of STAT3 knockdown in UM-SCC-1 ALDH^{high}CD44^{high} cells. **(A)** STAT3 knockdown efficiency of three shRNA-STAT3 constructs in UM-SCC-1. shRNA-STAT3 construct 3 was used for further study. **(B)** Bar graph depicts number of orospheres generated from shRNA-STAT3 UM-SCC-1. **(C)** FACS analysis of the ALDH^{high}CD44^{high} cell population between shRNA-control and shRNA-STAT3 UM-SCC-1.

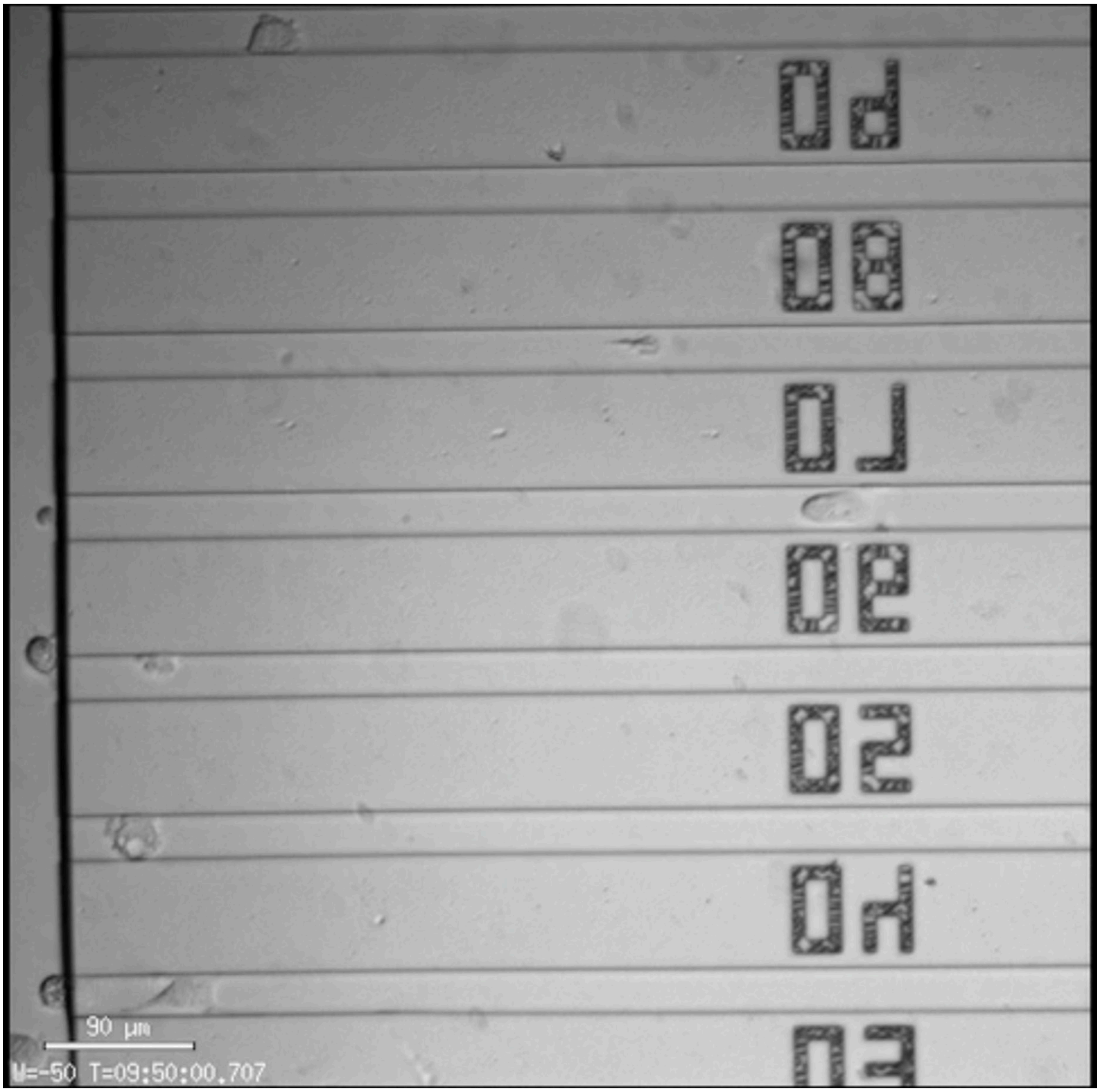

Supplementary Video 1: Timelapse images of ALDH^{high}CD44^{high} cells (sorted from UM-SCC-1) migrating towards the chemotactic gradient generated by primary human endothelial cells (HDMEC) in a microfluidics device.

See Supplementary Video 1
